# Targeting SR-BI for Cancer Diagnostics, Imaging and Therapy

**DOI:** 10.3389/fphar.2016.00326

**Published:** 2016-09-27

**Authors:** Maneesha A. Rajora, Gang Zheng

**Affiliations:** ^1^Princess Margaret Cancer Centre and Techna Institute, University Health NetworkToronto, ON, Canada; ^2^Institute of Biomaterials and Biomedical Engineering, University of TorontoToronto, ON, Canada; ^3^Department of Medical Biophysics, University of TorontoToronto, ON, Canada

**Keywords:** scavenger receptor BI, cancer imaging, cancer therapy, biomarkers, HDL, nanomimetics

## Abstract

Scavenger receptor class B type I (SR-BI) plays an important role in trafficking cholesteryl esters between the core of high density lipoprotein and the liver. Interestingly, this integral membrane protein receptor is also implicated in the metabolism of cholesterol by cancer cells, whereby overexpression of SR-BI has been observed in a number of tumors and cancer cell lines, including breast and prostate cancers. Consequently, SR-BI has recently gained attention as a cancer biomarker and exciting target for the direct cytosolic delivery of therapeutic agents. This brief review highlights these key developments in SR-BI-targeted cancer therapies and imaging probes. Special attention is given to the exploration of high density lipoprotein nanomimetic platforms that take advantage of upregulated SR-BI expression to facilitate targeted drug-delivery and cancer diagnostics, and promising future directions in the development of these agents.

## Introduction

Upregulation of cell-surface receptors in malignant tissue is both a hindrance and an opportunity in cancer therapy. On one hand, receptor upregulation enhances cancer cell response to growth factors, enabling one of their defining characteristics of self-sufficient proliferation (Hanahan and Weinberg, 2000). However, these overexpressed cell-surface receptors can also be opportunistically used to target cancer therapies. This is a highly desirable treatment strategy with the potential to enhance therapeutic indices by confining antineoplastic and cytotoxic effects to tumors. To this end, scavenger receptor class B type I (SR-BI) has recently been pursued as a target to facilitate cancer therapy and imaging.

SR-BI is an integral membrane glycoprotein receptor that plays a crucial role in the metabolism of high-density lipoprotein (HDL; Krieger, 1999). SR-BI binds HDL with high affinity to mediate selective cellular uptake and efflux of cholesteryl esters from the lipoprotein core (Acton et al., 1996; Ji et al., 1997). This uptake is made highly efficient via the formation of a non-aqueous channel that permits the direct cytosolic influx of the lipid core with no corresponding lysosomal degradation (Rodrigueza et al., 1999). In addition to mediating the transfer of cholesterol between HDL and healthy cells (predominantly within the liver and steroidogenic tissue), SR-BI also facilitates the selective uptake of cholesterol by malignant cells. As summarized in **Table 1**, several patient tumors and cancer cell lines display upregulated expression of SR-BI relative to healthy tissue, rendering SR-BI an interesting cell-surface receptor to pursue for targeted cancer therapy and imaging. In this mini review, we highlight recently explored strategies that take advantage of SR-BI overexpression and its capacity for direct cytosolic delivery of HDL cargo for the development of targeted cancer drug-delivery systems, imaging probes, and biomarkers.

**Table 1:** SR-BI endogenous ligands, expression in healthy tissue, upregulation in patient derived tumor samples and expression by cancer cell lines (OxLDL, AcLDL, and LPS represent oxidized LDL, acetylated LDL and lipopolysaccharides, respectively).

Endogenous ligands	Healthy tissue	Patient tumor samples	Cancer cell lines
HDL LDL VLDL OxLDL AcLDL Lipid-free apolipoproteins (apoA-I, apoA-II, apoC-II, apoE2, apoE3, apoE4) Serum amyloid A LPS Vitamin E (Calvo et al., 1997; Xu et al., 1997; Liadaki et al., 2000; Thuahnai et al., 2003; Vishnyakova et al., 2003; Baranova et al., 2005; Reboul et al., 2006)	Liver Steroidogenic tissues (testis, ovaries, adrenals) Brain Small and large intestine Platelets (Acton et al., 1996; Husemann and Silverstein, 2001; Lobo et al., 2001; Imachi et al., 2003)	Adrenal tumors Breast cancer Lymphoma Nasopharyngeal carcinoma Pancreatic cancer Prostate cancer Prostate bone metastasis Testicular cancer (Imachi et al., 1999; Arenas et al., 2004; Cao et al., 2004; Thysell et al., 2010; Yang et al., 2013; Zheng et al., 2013; Schorghofer et al., 2015; Julovi et al., 2016; Yuan et al., 2016)	***Adrenocortical:*** NCI-H295R ***Brain:*** SK-N-MC, MC-IXC, SK-KN-DW, 1321N1, U87 ***Breast:*** MDA-231, MDA (MB) 231, MCF7, P-148, Bone, 361, T4TD, T470, SK-Br-3 ***Colorectal:*** RKO, SWA480, SW620, HCT116, IGROV ***Epithelial:*** KB ***Ovarian:*** HIO-160, HeyA8, HeyA8-MDR, SKOV3ip1, OV 1063 ***Leukemia:*** THP-1,MT2, NB4, NB4 0076/6, MT1 ***Liver:*** HepG2, HUH-7, HF1 ***Lung:*** A549 ***Lymphoma:*** LY3, SUDHL-4, SUDHL-6, Farage, Ramos, Farage, Raji, Namalwa, Daudi, Jeko, HH, Hut-78 ***Nasopharyngeal***: 6-10B, SUNE1, CNE2, CNE1, SUNE2, 5-8F, ***Pancreatic:*** Aspc1, L3-6PL, Panc48, Panc1, MIAPaCa-2, CFPAC-1 and BxPC3 ***Prostate:*** 22RVI, PC3, LnCap, DU145 ***Testicular:*** R2C (Lacko et al., 2002; Rao, 2002; Pilon et al., 2003; Wadsack et al., 2003; Cao et al., 2009; Fletcher et al., 2010; Shahzad et al., 2011; Shin et al., 2012; Yang et al., 2013; Zheng et al., 2013; Julovi et al., 2016)

## SR-BI Targeting Strategies for Cancer Therapy

The ability of SR-BI to mediate direct cytosolic transport of HDL cholesteryl esters is a particularly lucrative characteristic to exploit for drug-delivery, as it overcomes reliance on endo-lysosomal uptake routes that lead to drug degradation. HDL-nanomimetics, composed of phospholipids and the most abundant HDL apolipoprotein, A-I (apoA-I), have thus been designed to exploit this cytosolic drug-delivery pathway. As reviewed elsewhere (Ng et al., 2011; Thaxton et al., 2016), HDL-nanomimetics possess many traits suited for drug-delivery, including lengthy circulation half-lives, stable core-loading with hydrophobic drugs, surface-functionalization by cholesterol-conjugated agents, and a sub-30 nm size favorable for enhanced nanoparticle permeation through tumor extracellular matrices. Furthermore, reviews of clinical HDL-infusion therapies demonstrate that elevations in plasma HDL levels (up to 30-fold) are well-tolerated following HDL-nanomimetic administration at doses of 0.25–135 mg/kg, whereby the nanoparticles subsequently participate in endogenous HDL receptor binding and metabolic pathways of interest for drug-delivery and atherosclerosis therapy, including reverse cholesterol transport leading to remodeling and equilibration of the administered HDL into spherical nanoparticles (Kingwell and Chapman, 2013; Simonsen, 2016). To this end, HDL-nanomimetics have been explored for SR-BI-mediated delivery of chemotherapeutics, small interfering ribonucleic acid (siRNA), and photosensitizers, as summarized in **Table 2**. Although lipoprotein-mimetic platforms have been widely explored more generally for cancer theranostics, this review focuses on agents that were studied to specifically target SR-BI.

**Table 2:** Examples of SR-BI-targeted drug and imaging probe delivery vehicles applied to *in vitro* and *in vivo* cancer models.

Platform	Drug or probe delivered	*In vitro* models	*In vivo* models	Reference
**Chemotherapy delivery**				
ApoA-I rHDL	Paclitaxel	Breast	-	(Lacko et al., 2002;
		Colon		McConathy et al., 2008;
		Ovarian		Mooberry et al., 2010;
		Prostate		Lee et al., 2015)
	Doxorubicin	Liver	Metastatic liver model^∗^	(Yuan et al., 2013)
	Epothilone B	Breast	-	(Lee et al., 2015)
		Ovarian		
		Colon		
Plasma HDL	RRR-α-tocopheiyl-succinate	Lung	-	(Hrzenjak et al., 2004)
HPPS	Paclitaxel oleate	Epithelial	Epithelial	(Yang et al., 2011a)
**siRNA delivery**				
ApoA-I rHDL	STAT3 siRNA	Ovarian^∗^	Ovarian^∗^	(Shahzad et al., 2011)
		Drug resistant ovarian^∗^	Drug resistant ovarian^∗^	
		Colon	Colon	
	VEGF siRNA	Breast	Breast	(Ding et al., 2014)
HPPS	blc-2 siRNA	Epithelial	Epithelial	(Yang et al., 2011b; Lin et al., 2012)
Gold-templated rHDL	VEGFR2 siRNA	-	Lung	(Tripathy et al., 2014)
**Dual targeting**				
ApoA-I rHDL	Gambogic acid (STM: pH-responsive cell penetrating peptide) Dichloroacetate (STM: sigma receptor-targeted anisamide)	Liver Lung	Liver Lung	(Ding et al., 2015) (Zhang et al., 2016)
HPPS	Ovalbumin antigen	-	Lymphoma	(Qian et al., 2016)
			Melanoma	
	DiR-BOA dye (STM: EGF to target EGFR)	Epithelial	Epithelial	(Zhang et al., 2010)
		Lung	Lung	
**Imaging**				
HPPS	Bacteriochlorin-BOA	-	Epithelial	(Cao et al., 2009)
	DiR-BOA and bcl-2 siRNA	Prostate	Prostate^∗^	(Lin et al., 2014b)
	^64^Cu-Porphyrin-lipid	-	Prostate^∗^	(Cui et al., 2015)
Calcium carbonate templated rHDL	Methylene blue	Lung	Lung	(Lu et al., 2015)

*Unless stated, all in vivo models are heterotopic xenografts (STM: second targeting moiety, (^∗^): orthotopic model).*

### Delivery of Chemotherapeutics

The first reports of SR-BI-targeted drug-delivery vehicles for cancer therapy aimed to enhance therapeutic effects and reduce off-target toxicity of chemotherapeutics. In 2002, Lacko et al. generated a paclitaxel-loaded apoA-I-reconstituted HDL (rHDL) vehicle that underwent uptake by various SR-BI-expressing cancer cell lines, including human prostate DU145 and PC3 cells (Lacko et al., 2002). Encapsulation of cargo within apoA-I-rHDL delayed its degradation in serum and facilitated SR-BI-selective cargo delivery (McConathy et al., 2011). In follow-up studies, this uptake resulted in a five-fold enhancement in paclitaxel delivery to OVAR-3 cells and 5- to 20-fold enhancement in cytotoxicity against ovarian, prostate and breast cancer cells versus free paclitaxel (McConathy et al., 2008; Mooberry et al., 2010). This enhanced *in vitro* therapeutic efficacy was coupled with increased *in vivo* tolerance of the paclitaxel-rHDL formulation relative to free paclitaxel or the clinically approved Abraxane^®^ albumin-paclitaxel nanoformulation (McConathy et al., 2008). ApoA-I-rHDL nanoconstructs were similarly used by other researchers to facilitate SR-BI-targeted delivery of paclitaxel and other hydrophobic agents such as RR-α-tocopheryl-succinate and epothilone B to adeno, breast and lung cancer cells (**Table 2**). In each case, drug-rHDL treatment increased SR-BI^+^ cancer cell cytotoxicity versus free drug with the added benefit, as reported by Ding et al. (2014), of diminishing undesired cytotoxicity against cells with limited SR-BI expression. Interestingly, this SR-BI-targeting strategy was also extended to the encapsulation and delivery of the hydrophilic drug, doxorubicin (Yuan et al., 2013), wherein the efficient (>70%) loading of drug within rHDL halved its IC_50_ in hepatocellular carcinoma (HCC) cells, yielded sustained drug release, and reduced tumor size in an apoA-I-dependent manner.

One concern associated with the clinical translation of SR-BI-targeted apoA-I-rHDL delivery platforms is the necessity of apoA-I protein for particle functionalization. Apo-AI is isolated from human plasma or derived from bacterial recombinant protein expression, and consequently may be prone to low collection yields, batch-to-batch variability, and contamination. To this end, our group introduced HDL-mimicking-peptide-phospholipid nanoscaffolds (HPPS). These HDL-nanomimetics are built with an 18 residue apoA-I-mimetic α-helical amphipathic peptide, which similarly to apoA-I, constrains the particle size below 30 nm and directs the selective cytosolic delivery of core-loaded cargo to SR-BI^+^ cells both *in vitro* and *in vivo* (Zhang et al., 2009; Lin et al., 2014a). When core-loaded with paclitaxel oleate (PTXOL), HPPS suppressed tumor growth selectively in SR-BI^+^ lesions to the same extent as PTXOL, but unlike the free drug, exerted no significant tumoricidal effects in non-target SR-BI^-^ tumors (Yang et al., 2011a). Combined with an absence of incurred acute liver toxicity, these results demonstrate that HDL-nanomimetics such as HPPS are a suitable and therapeutically effective strategy to attenuate off-target toxicity via SR-BI-homing.

### Direct Cytosolic Delivery of siRNA

Perhaps one of the most promising therapeutic utilities of SR-BI-mediated cytosolic drug-delivery is stable transfection of cancer cells with siRNA. As widely reviewed (Devi, 2006; Oh and Park, 2009; Petrocca and Lieberman, 2011; Zuckerman and Davis, 2015), RNA interference (RNAi) is an intriguing cancer therapy whereby short double-stranded RNA interacts with complimentary messenger RNA within the cell cytoplasm for sequence-specific post-transcriptional silencing of target oncogenes. Barriers to successful RNAi therapy include rapid siRNA degradation in circulation, off-target accumulation, toxicity, inefficient intracellular delivery of siRNA complexes, and endosomal escape (Whitehead et al., 2009; Pecot et al., 2011). Conversely, SR-BI-facilitated siRNA delivery to cancer cells has several advantages. Cholesterol-conjugated siRNA readily binds to HDL, which mediates its cellular uptake *in vivo* via SR-BI (Wolfrum et al., 2007). This allows for endosome-independent, direct cytosolic siRNA delivery, significantly enhancing siRNA transfection and reducing *in vitro* and *in vivo* target protein expression in SR-BI^+^ cancer cells relative to free siRNA and on par with liposomal-siRNA complexes, which are the current clinical gold standards for mediating siRNA delivery *in vivo* (Yang et al., 2011b; Lin et al., 2012; Ding et al., 2014; Tripathy et al., 2014; Zuckerman and Davis, 2015). This SR-BI-mediated siRNA delivery has been conducted via apoA-I-rHDL, HPPS, and gold-templated HDL-nanomimetics to silence oncogenes involved in cell proliferation, differentiation, anti-apoptotic pathways, and angiogenesis *in vitro* and *in vivo* models of breast, epidermal, lung, ovarian, and colorectal cancer (**Table 2**). Encapsulation of siRNA within SR-BI-targeted nanoparticles delayed its degradation and extended its circulation half-life (Lin et al., 2012; Ding et al., 2014; Tripathy et al., 2014). Furthermore, SR-BI-targeting of siRNA yielded potent *in vivo* effects. In metastatic and taxane-resistant models of ovarian cancer, rHDL-mediated siRNA delivery reduced tumor burden by 60%, and when combined with chemotherapy decreased metastatic lesions by 86% and tumor growth by over 90% relative to controls (Shahzad et al., 2011). Tripathy et al. (2014) demonstrated potent inhibition of neovascularization and tumor growth in a Lewis lung carcinoma model using gold-templated HDL-nanomimetics, while Ding et al. (2014) observed similar effects in a breast cancer model with the use of apoA-I-rHDL. Importantly, *in vivo* therapeutic effects of bcl-2-siRNA-HPPS demonstrated by Lin et al. (2012) in KB tumor models coincided with an absence of acute off-target toxicity. Though further *in vivo* investigation of SR-BI-targeting, longitudinal safety, and therapeutic efficacy must be conducted, these preliminary studies showcase the exciting potential of SR-BI-mediated siRNA cancer therapy.

### Combining SR-BI-Homing with Alternative Drug-Delivery Strategies

Despite the successes described thus far in targeting SR-BI to enhance and confine cancer therapy to target tumor tissue, associated biodistribution data demonstrated significant off-target accumulation of drugs, particularly in the liver and spleen (Zhang et al., 2009; Yang et al., 2011a; Tripathy et al., 2014); an unsurprising observation given the abundant expression of SR-BI in normal hepatic and steroidogenic tissue (**Table 1**). Thus, to limit potential drug-induced systemic toxicity and to further amplify and target therapeutic activity to tumor tissue, the following strategies combining SR-BI-homing with additional targeting functionality were recently explored:

(1) Photosensitive porphyrin nanoparticles: Porphyrins are non-toxic, naturally occurring heterocyclic molecules that require activation by near infrared (NIR) light to generate cytotoxic reactive oxygen species for cancer photodynamic therapy. When formulated with porphyrin-lipid, HDL-nanomimetics can thus exhibit multidimensional tumor targeting via apoA-I/SR-BI interactions, site-specific laser irradiation, and the inherent cancer cell affinity displayed by porphyrins (Zheng et al., 2007). This was demonstrated by Ng et al. (2013), who showed that porphyrin-HDL nanodisc-induced cytotoxicity required both cellular SR-BI expression and laser light.
(2) pH-sensitive nanoparticles: The acidic microenvironment of tumors has been employed to enhance drug accumulation at target lesions in combination with SR-BI targeting. By functionalizing the surface of apoA-I-rHDL with pH-sensitive cell-penetrating peptides, Ding et al. (2015) demonstrated enhanced SR-BI-mediated uptake of the hydrophobic apoptosis inducer gambogic acid within the cytosol of HCC cells at a pH of 6.4 versus a normal physiological pH of 7.4.
(3) Dual receptor-targeted agents: The incorporation of a second targeting vector directed toward upregulated cell-surface receptors in malignant cells, such as the sigma receptor or epidermal growth factor receptor, was also explored to enhance the targeting specificity of SR-BI-directed therapies. Compared with singularly targeting SR-BI, this multi-receptor-targeted approach increased drug accumulation in cancer cells *in vitro* and tumor xenografts *in vivo*, and was shown to enhance the dichlororacetate/p53-induced suppression of tumor growth in a model of lung adenocarcinoma (Zhang et al., 2010, 2016).
(4) Antigen-mediated immunotherapy: HPPS was formulated with a fusion peptide consisting of α-helical apoA-I-mimetic peptide and an antigen peptide against ovalbumin (expressed by E.G7 lymphoma cells) to generate a lymphoma nanovaccine, which demonstrated the most potent *in vivo* therapeutic effects of the four multi-homing strategies described herein. The particles targeted SR-BI^+^ mature dendritic cells, augmenting antigen uptake *in vivo* by up to 100-fold relative to free antigen delivery. This increased the ability of dendritic cells to augment CD8^+^ T-cell populations *in vivo*, allowing for immunization against lymphoma tumor growth, which was completely inhibited in a synergistic fashion when particles were further functionalized with cholesterol-conjugated toll-like receptor agonist CpG2395. This ultimately led to a survival rate of 83 %, whereas free fusion peptide was associated with complete mortality.

The positive findings from these preliminary studies support further evaluation of SR-BI-facilitated multi-targeted nanomedicines for cancer therapy, including complete characterization of particle biodistribution and therapeutic efficacy in orthotopic animal cancer models.

### Antagonizing SR-BI Activity

Dysregulation of cholesterol metabolism has been associated with tumor growth since the early 1900’s, whereby the proliferation and migration of cancer cells is thought to require increased cholesterol influx, yielding higher cellular cholesterol levels in malignant tissue (Swyer, 1942; Dessì et al., 1994; Thysell et al., 2010; Cruz et al., 2013; de Gonzalo-Calvo et al., 2015). SR-BI was shown to play a role in this process, such that its inhibition reduced cancer cell proliferation (Pussinen et al., 2000; Cao et al., 2004; Leon et al., 2010; Julovi et al., 2016). Thus, a few recent reports sought to therapeutically mitigate the pro-tumorigenic activity of SR-BI. Twiddy et al. (2012) demonstrated that siRNA knockdown of SR-BI in castration-resistant prostate cancer cells significantly decreased cell viability and secretion of prostate-specific antigen, a biomarker of prostate cancer. Subsequently, Yang et al. (2013) designed 13 nm gold-templated, apoA-I-functionalized HDL-nanomimetics that selectively inhibited SR-BI^+^ B-cell lymphoma growth by attenuating cholesterol influx into SUDHL-4 lymphoma cells, while displaying no apoptosis induction in human primary hepatocytes and macrophages. HPPS was similarly shown to attenuate tumor growth in a nasopharyngeal carcinoma animal model, which the authors proposed via *in vitro* studies to be a result of inhibiting SR-BI-regulated cell motility (Zheng et al., 2013). The findings from these studies stand in line with the known protective effects of ApoA-I and HDL on tumor growth (Su et al., 2010; Zamanian-Daryoush and DiDonato, 2015), presenting an alternative and less explored strategy of enforcing SR-BI-directed cancer therapy.

## SR-BI-Mediated Cancer Imaging and Diagnostics

The overexpression of SR-BI in patient tumor samples relative to healthy tissue also potentiates its use for cancer imaging and diagnostics. Although many of the SR-BI-targeted nanoparticle drug-delivery systems discussed above were formulated with NIR dyes for biodistribution analyses, limited studies have actively pursued SR-BI-targeting for the generation of cancer imaging probes. For example, in 2009, Cao et al. developed apoA-I-rHDL core-loaded with bis-oleate-functionalized bacteriochlorin (BChl), a fluorescent dye excitable by 750–850 nm NIR light; a range that falls within the optical window optimal for fluorescence imaging *in vivo* with minimal absorbance interference from hemoglobin and water (Cao et al., 2009). The authors demonstrated SR-BI-selective cellular uptake of the BChl core *in vitro*, which translated into strong contrast visualized with a small animal fluorescence imaging system in KB tumor tissue *in vivo*. ApoA-I HDL-mimetics were also core-loaded with methylene blue for apoA-I-mediated *in vivo* NIR fluorescence imaging of SR-BI^+^ lung carcinoma xenografts (Lu et al., 2015). Core-loading of HPPS with NIR fluorophores was also conducted to image the biodistribution of co-loaded siRNA for treatment planning purposes (Lin et al., 2014b). By monitoring fluorescence contrast at tumor sites, the authors were able to select a dose regimen that confined particle accumulation and potent apoptotic effects of the siRNA therapy to the target lesion. This selective fluorescence contrast was further proposed by the authors to have utility in image-guided tumor resection. In addition to fluorescence imaging, SR-BI-targeted HPPS was also amenable to generating contrast for positron emission tomography imaging of deep-seated orthotopic prostate tumors when formulated with a ^64^Cu-porphyrin-lipid shell (Cui et al., 2015). It should be noted that the tumor-homing displayed by these particles is likely contributed to by the enhanced permeability and retention (EPR) effect, hallmarked by the increasing contrast observed at the tumor site over a 24–48 h time period following agent administration. Consequently, further *in vivo* characterization of selective particle uptake must be conducted to ensure SR-BI-specificity is maintained by the contrast agents for accurate diagnostic and imaging purposes.

The upregulated expression of SR-BI in itself has been proposed to serve as a biomarker for tumor malignancy. The histological analysis of 106 prostate cancer biopsies by Schorghofer et al. (2015) revealed a positive correlation between elevated SR-BI expression and tumor grade, metastasis, and poorer patient outcomes. Similarly, higher SR-BI expression in breast cancer tissue was associated with increased disease aggressiveness and patient mortality (Yuan et al., 2016), while RNA microarrays of samples from patients with chronic myeloid leukemia identified a six gene profile including *SCARB1* (the gene encoding for SR-BI) that discriminated early and late-stage disease (Oehler et al., 2009). These promising initial studies thus suggest that SR-BI may be a viable biomarker of cancer prognosis. Nevertheless, due to the ubiquitous expression of SR-BI in malignant and healthy tissue, key consideration of the specificity associated with the use of SR-BI as a biomarker will be imperative in assessing its prognostic value.

## Summary and Perspectives

The overexpression of SR-BI in malignant tissue has been exploited for the development of targeted cancer therapies, imaging probes, and prognostic biomarkers. SR-BI-targeted vehicles can mediate the selective transfer of drugs from HDL-mimetics into the cytosol of malignant cells, which is particularly valuable for enhancing siRNA delivery efficiency for cancer gene therapy. Although promising *in vivo* therapeutic effects were demonstrated by the SR-BI-directed therapies overviewed herein, a number of key challenges must be addressed to advance the utility of what is still a relatively unexplored targeting strategy. Firstly, the ubiquitous expression of SR-BI in healthy tissue, and particularly its abundant expression in the liver and steroidogenic organs, presents a challenge in assuring the *in vivo* specificity of SR-BI-targeted agents. As such, careful consideration is due when exclusively targeting SR-BI for the delivery of chemotherapeutics with non-specific mechanisms of action, especially those which may cause liver toxicity. To this end, it is imperative that researchers expand upon the currently limited evaluation of acute and chronic systemic toxicity of SR-BI-targeting agents. Furthermore, quantification of drug-delivery to tumors and major organs and the unequivocal demonstration of SR-BI targeting *in vivo* (for example via competitive inhibition and comparative studies in SR-BI^+^ and SR-BI^-^ tumor models) should become mainstays within the field. In order to obtain more clinically relevant information, these evaluations should extend beyond typically used heterotopic tumor models to include orthotopic models. The reliance on inherently disrupted tumor vasculature and the EPR effect for tumor accumulation of the explored HDL-nanomimetics also creates a barrier to efficient SR-BI-targeted cancer therapy. Though widely used as a targeting strategy in nanomedicine development, the clinical relevance of the EPR effect is disputed as it does not accurately represent the vasculature and microenvironment of many human tumors (Gillies et al., 1999; Nichols and Bae, 2012). Strategies that target nanoparticle extravasation to tumor sites without requiring inherently disrupted tumor vasculature to facilitate delivery may thus enhance the delivery efficiency and therapeutic relevance of SR-BI-targeted HDL-mimetics. For example, Mulik et al. (2016) employed microbubble-enhanced focused ultrasound to selectively disrupt the blood-brain barrier in rats to enhance the site-specific delivery of lipoprotein-mimetics to target brain tissue. Additionally, given the multi-ligand status of SR-BI (**Table 1**), ample opportunity exists for the exploration of alternative targeting ligands to apoA-I, which may access a wider variety of tumors and alter particle distribution profiles. Ultimately, with further exploration of alternative SR-BI-targeting vectors and more rigorous *in vivo* characterization of existing delivery platforms, a better understanding of the utility of SR-BI-targeting for cancer therapy and imaging will be gained.

## Author Contributions

MR and GZ were involved in the conception, drafting, and editing of the manuscript to ensure an accurate review of literature.

## Conflict of Interest Statement

The authors declare that the research was conducted in the absence of any commercial or financial relationships that could be construed as a potential conflict of interest.
